# Development and Validation of a HPV-32 Specific PCR Assay

**DOI:** 10.1186/1743-422X-6-90

**Published:** 2009-06-27

**Authors:** Nicholas R Herrel, Nadia L Johnson, Jennifer E Cameron, Janet Leigh, Michael E Hagensee

**Affiliations:** 1 Department of Microbiology, Immunology, and Parasitology, Louisiana State University Health Sciences Center, New Orleans, USA; 2 Department of Medicine, Louisiana State University Health Sciences Center, New Orleans, USA; 3 Department of Oral Medicine, Louisiana State University Health Sciences Center, New Orleans, USA; 4 Cancer Center, Tulane University Health Sciences Center, New Orleans, USA

## Abstract

**Background:**

Human Papillomavirus-32 (HPV-32) has traditionally been associated with focal-epithelial-hyperplasia (FEH). It is also present in 58% of oral warts of HIV-positive individuals whose prevalence is increasing. Current methods for the detection of HPV-32 are labor-intensive and insensitive so the goal of this work was to develop a highly sensitive and easy to use specific polymerase chain reaction (PCR) assay.

**Materials and methods:**

An HPV-32 L1 specific PCR assay was developed and optimized. The sensitivity and specificity was compared to previous assays utilized for detection (PGMY and MY09/11 PCR with dot blot hybridization) using cloned HPV-32 L1, the closely related HPV-42 L1 as well as clinical samples (oral swabs and fluids from 89 HIV-positive subjects).

**Results:**

The HPV-32 specific PCR assay showed improved sensitivity to 5 copies of HPV-32 as compared to the PGMY PCR, MY09/11 PCR and dot blot which had a limit of detection of approximately 3,000 copies. Using the HPV-32 dot blot hybridization assay as the gold standard, the HPV-32 specific PCR assay has a sensitivity of 95.8% and 88.9% by sample and subject, respectively, and specificity was 87.8% and 58.8% by sample and subject, respectively. The low sensitivity is due to the HPV-32 specific PCR assays ability to detect more HPV-32 positive samples and may be the new gold standard.

**Conclusion:**

Due to the ease, sensitivity, and specificity the HPV-32 specific PCR assay is superior to previous assays and is ideal for detection of HPV-32 in large cohorts. This assay provides an excellent tool to study the natural history of HPV-32 infection and the development of oral warts.

## Background

Human Papillomavirus (HPV) is the most common sexually transmitted viral infection in the world with greater than 100 genotypes described to date [1-3]. High risk HPV genotypes (HPV-16 and -18 for example) are associated with human malignancy including as much as 95% of cervical cancers and up to 35% of oral malignancies. The low risk HPV types, for example HPV-6 and -11, are the etiologic agent of benign hyperproliferations (warts) with can occur in the genital tract or oral cavity and are a significant health problem.

HPV-32 has only been well described in the oral cavity. In a study of 67 high-risk individuals composed of more than 85% HIV-positive individuals HPV-32 was the most prevalent type detected in oral lesions (40%)[4]. It was detected in 30% of warts, 67% of FEH, 50% of fibroma, and 20% of focal keratosis. This study utilized a complex series of PCR assays designed to detect a broad spectrum of HPV types including genital, oral, and cutaneous types found in humans as well as animal. This was accomplished utilizing a series of primers for highly conserved sequences in the L1 open reading frame and radiolabeled probes for Southern blot analysis. In a New Orleans HIV-positive cohort 58% of oral wart biopsies contain HPV-32 DNA with HPV-6, -7, -11, -16, -18, and -73 also detected [5]. HPV-32 was detected by amplification with degenerate MY09/11 primers designed to detect genital HPV types followed by direct sequencing. In the advent of widespread use of highly active anti-retroviral therapy (HAART) an increase in oral warts has been observed while decreases in other types of oral lesions have been noted over the same time period [6-8]. This coupled with the fact that most oral warts in HIV-positive individuals are due to HPV-32 makes further study into the natural history of these HPV-32 infections warranted.

The prevalence rate of HPV-32 infection in the oral cavity of HIV-positive and HPV-negative populations without obvious lesions has not been well studied. One study did show a prevalence rate of 1.6% in a cohort of normal, healthy individuals from South Africa [9] utilizing MY09/11 degenerate primers followed by direct sequencing. Using the same primers in conjunctions with dot-blot hybridization, Cameron and Hagensee showed a prevalence rate of 5.7% in the New Orleans HIV-positive cohort [5]. This assay was shown to be rather insensitive and able to only detect 2.5 picograms of cloned HPV-32 DNA [5].

The New Orleans and South African studies described above used PCR assays utilizing primers designed to detect genital HPV types (MY09/11 and PGMY primer sets). Although these methods are capable of detecting HPV-32, the sensitivity has not been determined but is expected to be low. With an increase in oral HPV infection and disease in HIV-positive individuals on HAART, and with HPV-32 being prevalent in the oral cavity, improved methods to detect HPV-32 are warranted. This study describes the development of a type-specific PCR-based assay to detect HPV-32 for the application in a natural history study of HPV-32 and oral wart progression.

## Results

### Sensitivity of the Current HPV Detection Assays

**Figure 1:**
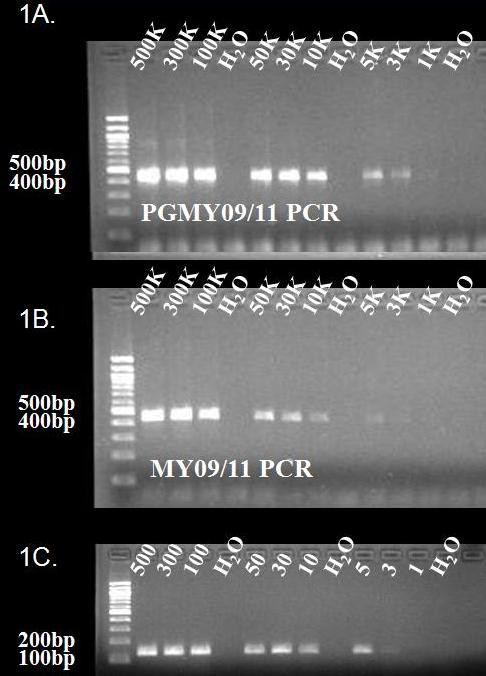
**Sensitivity of the PGMY09/11 (1A), MY09/11 (1B), and HPV-32 Specific (1C) PCRs were tested using dilutions of plasmid (pMJ32L1) containing HPV-32 L1 gene**. Each assay was tested on a dilutions scheme starting at 500,000 down to 1 copy. Figure contains a representative gel from 6 experiments (three separate dilutions done in duplicate). The sensitivity of both the PGMY09/11 and MY09/11 PCR assays were shown to be approximately 3,000 copies of HPV-32 L1. The HPV-32 Specific PCR consistently detected 5 copies of HPV-32 L1.

**Figure 2:**
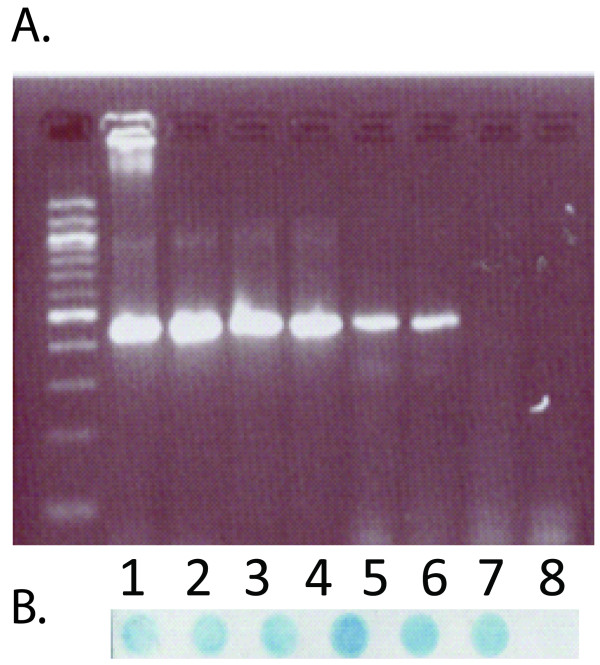
**Sensitivity of the HPV-32 dot blot assay**. (A) Serial dilution of pMJ32L1 detected via MY09/11 PCR. (B) The hybridization step did not increase the sensitivity of the assay.

Previous work in our laboratory [5] has noted that both the PGMY and MY09/11 primer sets will amplify HPV-32 using either cloned HPV-32 DNA or DNA isolated from oral warts determined to be homologous to HPV-32. After amplification, the identity of the HPV types amplified was confirmed to be HPV-32 by dot-blot hybridization using specific HPV-32 probes. The sensitivity of both PCR amplification step and dot blot was never formally tested. The sensitivity of these primer pairs was tested by attempting to amplify serial dilutions of the cloned HPV-32 L1 gene (pMJ32L1). Three separate 10× dilution schemes were generated: 500,000 → 5; 300,000 → 3; and 100,000 → 1. These dilution series were each tested three times and each dilution scheme was run in duplicate for a total of 6 experiments. These two assays showed a comparable sensitivity with the ability to detect approximately 3,000 copies of HPV-32 L1 gene (Figure 1A, B). Hybridization steps can often increase sensitivity and this was tested utilizing the amplicons from the cloned HPV-32 L1 gene dilution series that were amplified with the MY09/11 primers. The addition of the dot blot hybridization step using HPV-32 specific probes did not affect the overall sensitivity of the assay (Figure 2). The Roche reverse line blot system does not utilize an HPV-32 specific probe in the line blot assay so sensitivity of this genotyping system could not be done. Due to this relative lack of sensitivity for both amplification systems, a HPV-32 type specific PCR assay was developed.

### HPV-32 Specific PCR Development

PCR primers were designed using PrimerSelect in the LaserGene 6 DNA and Protein Analysis Software (DNASTAR, Inc., Madison, WI) to generate a 100–200 bp amplicon and have no cross-reactivity with other known DNA sequences. The HPV-32 specific primers (HPV-32 Det For and Rev, table 1) generate a 134 bp amplicon and have no potential cross-reactivity with human genomic DNA and limited cross-reactivity with known HPV types as tested utilizing NCBI's Basic Local Alignment Search Tool (BLAST, ). The assay was optimized by varying the magnesium concentration and annealing temperature (data not shown). Due to the primers need for a precise magnesium concentration for optimal sensitivity a commercial master mix could not be utilized. To limit variation in PCR reactions and pipeting errors a master mix for each PCR was produced and aliquoted appropriately

**Table 1:** Summary of the HPV detection assays.

**Name**	**Primers**	**HPV**	**Amplify**	**Genotype**
PGMY	PGMY09/11	+/-	Most Genital Genotypes	N.A.
Reverse Line Blot Assay	PGMY09/11	+/-	N.A.	27 Genital Genotypes
HPV-32 Dot Blot System	MY09/11	+/-	Most Genital Genotypes	HPV-32
HPV-32 Specific PCR Assay	HPV-32 Det For/Rev	+/-	HPV-32	HPV-32

### Sensitivity

Serial dilutions of known quantities of cloned HPV-32 L1 (pMJ32L1) was used to determine the sensitivity of the HPV-32 specific assay. These experiments showed that the HPV-32 specific PCR was reproducibly sensitive down to 5 copies of the HPV-32 L1 gene (Figure 1C).

### Specificity

Specificity of any HPV PCR assay is of significant concern because genotypes are defined by only a 10% difference in the nucleotide sequence of their E6, E7, and L1 open reading frames (ORFs). For this reason a number of experiments were performed to test the assay's specificity. First, because of the potential of cross-priming, the primer's sequences were compared to other HPV types. This analysis noted that the HPV-42 L1 gene was the most closely related to HPV-32. HPV-42 has no mismatches in the forward primer and only four mismatches in the reverse primer. Purified and quantified pMJ42L1 (10^7 ^billion copies) was used as the template for four separate PCR reactions and in all cases, HPV-42L1 gene was not amplified, while only 5 copies of the HPV-32 L1 gene was detected (data not shown). Next, using clinical material, six archival samples that had previously tested positive for HPV-42 by the Roche reverse line blot assay were identified and retested positive for HPV-42. These six samples were then subjected to PCR amplification using the HPV-32 L1 specific primers. Two of the six samples amplified. The assay volume was quadrupled (100 μL), reamplified, and the resultant amplicons sequenced. They were found to have the best homology (85.4% and 98.7%) with HPV-32. This implied that this clinical sample contained both HPV-32 and HPV-42. Finally, clinical samples (45 subjects) that were previously found to contain HPV types (number): 6 (n = 5), 16 (8), 18 (2), 26 (10), 33 (1), 35 (2), 39 (19), 45 (21), 51 (1), 53 (2), 55 (17), 58 (5), 59 (13), 62 (6), 66 (3), 69 (1), MM4 (12), MM7/83 (24), MM8 (13), and MM9 (7) were amplified via the HPV-32 Specific PCR and were found to be negative.

### Reproducibility

Subjects (n = 89) were enrolled in a study on oral HPV infection from the New Orleans HOP clinic (see Materials and Methods). Reproducibility of the assay was assessed by retesting 14 patients (111 samples) with 57 of these samples positive for HPV-32. Overall, 94.6% of the samples were reproducible. Gingiva, tongue, sublingual, and saliva sites each had a single sample not reproduce, while the hard palate site had two samples not reproduce (table 2).

**Table 2:** Reproducibility of the HPV-32 Specific PCR with samples from 14 subjects

**Site**	**Reproduced/Total**	**Percent Reproduced**
Labial	13/13	100
Buccal Mucosa	14/14	100
Gingiva	11/12	91.7
Tongue	13/14	92.9
Sublingual	11/12	91.7
Hard Palate	11/13	84.6
Tonsils	12/12	100
Saliva	11/12	91.7
Gargle	9/9	100

### Comparison of the HPV-32 Specific PCR to the Dot Blot Assay

The optimized HPV-32 Specific PCR assay was compared to the laboratory's previous gold standard assay for detecting HPV-32: PCR amplification using MY09/11 primers followed by dot blot hybridization using HPV-32 specific probes. A group of 663 samples from 89 HIV-positive subjects who were screened positive for HPV DNA using PGMY primers but could not be genotyped using the reverse line blot assay. The integrity of these stored samples were verified by the detection of the β-globin gene. The HPV-32 dot blot assay detected HPV-32 in twenty-four oral samples (3.6%) from nine subjects (10%). All but one (23) of these HPV-32 positive samples were positive by the HPV-32 specific PCR (Table 3). An additional 78 HPV-32 positive samples were identified that were negative by the dot blot assay. The HPV-32 type specific PCR assay has a sensitivity of 95.8% and a specificity of 87.8% with a kappa of 0.32 ± 0.029 as compared to the HPV-32 dot blot assay. When analyzed according to subjects, one subject was not detected with the HPV-32 specific assay which was detected by the dot blot assay. In contrast, 33 subjects (37%) were positive by the HPV-32 specific assay and negative via the HPV-32 dot blot (kappa of 0.18 ± 0.068; sensitivity of 88.9%; specificity of 58.8%, Table 4).

**Table 3:** Comparison of the HPV-32 Specific PCR and MY09/11 HPV-32 dot blot detection methods by samples

		**HPV-32 PCR**	
		**+**	**-**

**HPV-32 Dot Blot**	**+**	23	1
	**-**	78	561

**Table 4:** Comparison of the HPV-32 Specific PCR and MY09/11 HPV-32 dot blot detection methods by subjects

		**HPV-32 PCR**	
		**+**	**-**

**HPV-32 Dot Blot**	**+**	8	1
	**-**	33	47

The new HPV-32 specific PCR system targeting the L1 gene demonstrated significantly increased sensitivity as compared to the laboratory's previous gold standard of MY09/11 amplification and dot blot hybridization. This could be due to the robustness of this new assay or due to laboratory contamination. Review of the PCR runs noted no false positive in no template controls making lab contamination less likely. A verification assay was developed which targeted the HPV-32 E6/E7 region. In a group of 45 oral samples from 5 patients, the E6E7 PCR assay confirmed 22 of the previously identified HPV-32 positive samples by the L1 assay. The E6E7 assay detected a positive sample among the 23 believed to be negative by the L1 assay. The agreement was almost perfect with a kappa of 0.96 ± 0.148. It is concluded that the increased sensitivity seen is due to the robustness of the HPV-32 L1 PCR assay implying that this assay is the new gold standard.

## Discussion

Previous studies in the Hagensee laboratory have shown HPV-32 to be the most common HPV type found in oral warts in HIV positive individuals[5]. The natural history of oral HPV-32 infection has not been extensively studied. In order to accurately characterize the natural history of this infection, a highly sensitive, rapid, inexpensive, and specific assay is required. The current method of detection of HPV-32 requires PCR amplification and a blot/probe hybridization which is labor-intensive and expensive. Utilizing purified pMJ32L1, the sensitivity for two gold-standard HPV PCR assays, PGMY09/11 and MY09/11 PCRs, was shown to be approximately 3,000 copies. In contrast, the PGMY09/11 Reverse Line Blot System can detect 10 to 1,000 copies of most of the genotypes found on the blot [10]. Utilizing an HPV-32 dot blot assay did not improve sensitivity, thus a HPV-32 type specific PCR assay was developed and optimized.

Sensitivity, specificity, and reproducibility are critical to the development of any new assay. The sensitivity of the HPV-32 assay was determined using purified HPV-32 DNA and was determined to be 5 copies of cloned DNA. This was significantly more sensitive than both the PGMY and MY09/11 based PCR amplification assays (3,000 copies). The superior sensitivity was confirmed with clinical samples whereby 33 subjects with a total of 78 samples that were negative by current detection assays were found to be positive for HPV-32 by the HPV-32 specific PCR assay. This increased sensitivity was confirmed by a second assay which detected a different gene in HPV-32. This sensitivity increase is predictable as it has been observed in genital HPV prevalence studies. A healthy non-immunosuppressed female population has genital HPV-16 infection rates varying from 2.0–5.4% using consensus/degenerate primers [11-13] while type specific primers detect higher rates of 9.4–9.7% [11-15].

An HPV genotype is defined as a ≥ 10% difference in E6, E7, and L1 open reading frame sequences. The specificity was tested theoretically by genomic alignments, as well as empirically and found to be excellent. Due to the higher sensitivity of the HPV-32 specific PCR, this HPV type was detected more readily in clinical samples; thereby, showing lower specificity, sensitivity, and agreement (kappa value) with the current detection methods. The issue of cross-reactivity was tested utilizing the most closely related DNA sequence known (HPV-42 L1) which has only four mismatches within the primer regions. The HPV-32 specific PCR did not amplify in the presence of > 10^7 ^copies of HPV-42 L1 thereby showing the stringent specificity of the PCR. Clinical samples positive for HPV-42 either did not have HPV-32 in them or were found to be co-infected with HPV-32. There was no evidence of cross-priming with the HPV-32 primers and amplification from a HPV-42 DNA template.

The reproducibility of the assay was shown by the ability to repeatedly detect HPV-32 in clinical samples. All oral cavity sites sampled showed high reproducibility (> 92%) with the exception of the hard palate (84.6%). The lower rate of reproducibility of the hard palate may be due to a low level of infection which would be at the level of detection of the HPV-32-specific PCR assay. This is supported by the relatively lower rate of infection (10.1%) at the hard palate and is also supported by the faint bands seen in 5 out of the 6 discordant results implying low viral loads.

Although this assay demonstrated increased sensitivity, excellent specificity and reproducibility, it is not optimal. Most modern PCR assays utilize a standardized universal master mix to which the specific primers are added. However, this approach does not allow one to vary the magnesium concentration which was critical to the optimization of the HPV-32 specific L1 assay in order to achieve the maximum sensitivity. The current assay could also be improved by the use of dUTP and addition of U-glucosidase step which would degrade any pre-existing amplicon DNA from previous amplification. This feature could further limit any additional laboratory contamination.

In conclusion, the HPV-32 specific PCR assay has been shown to have increased sensitivity, and excellent specificity as compared to current assays. In addition, this assay is highly reproducible and is less labor-intensive. This makes the assay ideal to assess the natural history of HPV-32 in the oral cavity.

## Materials and methods

### Cloning of HPV-32 and HPV-42 L1 Gene

HPV-32 and -42 L1 genes were cloned directionally into pMJ601 (designated pMJ32L1 and pMJ42L1, respectively), grown in *E. coli*, and extracted via a ProMega MiniPrep Kit according to their protocol. The DNA concentration was quantified using a DU 530 Life Sciences UV/Vis Spectrophotometer and confirmed by Eppendorf BioPhotometer.

### Patient Population

For the clinical samples utilized, subjects were enrolled in the Medical Center of Louisiana at New Orleans (MCLNO) HIV outpatient program (HOP) Clinic in New Orleans, LA, from May 2002 to December 2003. One-hundred-seventy subjects whose oral samples were previously tested for HPV DNA were utilized. Subjects gave informed consent by a trained professional according to the LSUHSC Institutional Review Board (IRB) and the MCLNO HOP research committee accepted protocol. All subjects were given a comprehensive exam for all oral lesions by a health professional. In addition to the oral samples, basic demographic information, peripheral CD4^+ ^T-cell count and HIV viral load was collected from each subject as well as current HIV medications.

### Sample Collection and Processing

Cells were collected from the buccal mucosa, labia, gingiva, tongue, sublingual surface, hard palate, and tonsils by vigorously rubbing the surfaces with a sterile swab (Puritan, Guilford, Maine). These sites were chosen for their association with HPV associated lesions and head and neck squamous cell carcinomas. The swabs were stored in Specimen Transport Medium (DIGENE, Gaithersburg, MD) at 4°C. Unstimulated expectorated saliva (5 mL) was collected in a 50 mL conical tube. Five milliliters of sterile saline was gargled and expectorated into a 50 mL conical tube. Both saliva and gargle samples were stored at 4°C as well. Saliva and gargle samples were processed within 4 hours, and sample DNA extracted within 18 hours of collection.

Saliva and gargle specimens were processed by homogenizing the sample through an 18 gauge needle and syringe. The specimen was then centrifuged at 1,260 × g for 10 minutes. Supernatants were removed, and the cell pellet was resuspended in 1 mL of sterile PBS and stored at 4°C until extraction.

DNA from all samples was extracted using the QIAGEN Blood Extraction Kit (Germany), according to the manufacturer's protocols. Extracted DNA was stored at -20°C until HPV detection by PCR was performed.

### HPV Genotyping

#### Reverse Line Blot System

HPV genotyping was done using the Roche HPV Reverse Line Blot system according to the manufacturer's protocol (Roche Molecular Systems, Inc., Alameda, California). Briefly, multiplex PCR amplified a 448 bp sequence of the L1 Gene and 268 bp sequence of β-Globin (housekeeping gene) using biotinylated PGMY09/PGMY11 consensus/degenerate primers and GH20/PCO4 primers, respectively [10]. Thermocycling was done on a PCT-100 (MJ Research, Inc Waltham, MA) as follows: 50°C for 2 minutes, 95°C for 9 minutes, 40 cycles of 95°C for 1 minute, 55°C for 1 minute, and 72°C for 1 minute, extension at 72°C for 5 minutes, followed by a 15°C hold. The amplicons were visualized on a 2.0% agarose gel with 0.5 μg/mL ethidium bromide. The PGMY primer amplification will detect most genital HPV types as well as HPV-32. A gel negative HPV result was defined as a sample demonstrating the 268 bp β-Globin band but no 448 bp HPV band. All HPV positive samples were applied to the reverse line blot system according to Roche's protocol. Briefly, the biotinylated PCR products were denatured for one hour at room temperature and hybridized at 53°C in a shaking water bath to strips containing probes for 27 HPV genotypes. (high risk: 16, 18, 26, 31, 33, 35, 39, 45, 51, 52, 55, 56, 58, 59, 68, MM4, MM7, and MM9; low risk: 6, 11, 40, 42, 53, 54, 57, 66, and MM8). The strips were then washed (1× SSPE-0.1% SDS) for 15 minutes in a 53°C shaking water bath. SA-HRP was added to the strips and shaken for 30 minutes at room temperature. This was followed by two 10-minute washes. The strips were incubated for 5 minutes, with shaking, in citrate buffer (0.1 M Sodium Citrate). Color development was accomplished via the addition of substrate 3,3',5,5'-tetramethylbenzidine (TMB) with hydrogen peroxide. A blue precipitate was observed at the probe locations that contained hybridized PCR products. The reaction was stopped after 5 minutes with the addition of water and read immediately.

#### HPV-32 Dot Blot System

Samples that were gel-positive and line blot-negative were then tested for HPV-32 via dot blot. This was performed by re-amplifying samples with the MY09/11 primers (table 1). The final concentrations in the 100 μL reaction were 1× PCR Buffer II, 1.0 μM of each primer (MY09 and MY11), 250 μM of each dNTP, 2 mM MgCl_2_, 7.5 U AmpliTaq™ Gold and 5 μL of template DNA. The thermocycler protocol was: 50°C for 2 minutes, 95°C for 9 minutes, 40 cycles of 95°C for 1 minute, 55°C for 1 minute, and 72°C for 1 minute, extension at 72°C for 5 minutes, followed by a 15°C hold. The 448 bp amplicons were detected and visualized as detailed above.

MY09/11 amplicons (10 μL) from positive patients were denatured for one hour in 100 μL of denaturation buffer (1.5 M NaCl, 0.5 M NaOH, 0.025 M EDTA). This mixture was applied to a nylon membrane (Micron Separations, Inc.) that was pre-moistened in 6× SSPE using a Dot Blot apparatus (Schleicher and Schuell, Keene, NH). The DNA was then UV crosslinked to the membrane in a UV Stratalinker 1800 (Stratagene, La Jolla, CA) using 120 mjoules and dried overnight. The nylon membrane was put in pre-hybridization buffer (0.1× SSPE-0.5% SDS) for one hour at 65°C, followed by a one hour incubation at 65°C in hybridization buffer (4× SSPE-0.5% SDS). The biotinylated HPV-32 probe (5'-Biotin-AGG TGC TGT TAC CTT AGC TTG-3') was mixed with hybridization buffer at a concentration of 0.5 pmol/mL, and the membrane was soaked in the probe solution for 30 minutes in a 53°C shaking water bath. The remaining procedure is done using the reverse line blot procedure as described above.

#### Specific Amplification of HPV-32 by PCR

The integrity of the stored DNA samples was verified via PCR amplification and the detection of the 268 bp amplicon of the β-globin gene. Final concentrations in the 25 μL reactions were 1× PCR Buffer II, 0.25 μM of each primer (GH20 and PC04; table 1), 200 μM of each dNTP, 4 mM MgCl_2_, 0.625 U AmpliTaq™ Gold and 5 μL of template DNA. Thermocycling protocol was performed as follows: 50°C for 2 minutes; 95°C for 9 minutes, 40 cycles of 95°C for 1 minute, 55°C for 10 seconds, and 72°C for 30 seconds, extension at 72°C for 5 minutes, followed by a 15°C hold. The 268 bp amplicons were detected and visualized as described above.

The primer sequences for the HPV-32 specific PCR were 5' GTG GCC GCC TAG TGA CAA C 3' (HPV-32 Det For) and 5' GAT GCC CAA CAG CCA AAA G 3' (HPV-32 Det Rev). The final concentrations for the 25 μL HPV-32 Specific PCR reaction were 1× PCR Buffer II, 0.5 μM of each primer, 200 μM of each dNTP, 1.5 mM MgCl_2_, 0.625 U AmpliTaq™ Gold and 5 μL of template. Thermocycling was performed as follows: 95°C for 9 minutes; 40 cycles of 95°C for 1 minute, 58.5°C for 10 seconds, and 70°C for 30 seconds; extension at 70°C for 5 minutes, followed by a 15°C hold. The 134 bp amplicons were detected and visualized as described above.

The primer sequences for the HPV-32 E6/E7 specific PCR were 5' TAT AAC GGA CGG CAT TTC AGA TTC 3' (HPV-32 E6E7 For) and 5' GTC ACT CCA CGC AGG CAC AC 3' (HPV-32 E6E7 Rev). The final concentrations for the 25 μL HPV-32 E6E7 Specific PCR reaction were 1× PCR Buffer II, 0.5 μM of each primer, 200 μM of each dNTP, 2.0 mM MgCl_2_, 0.625 U AmpliTaq™ Gold and 5 μL of template. Thermocycling was performed as follows: 95°C for 9 minutes; 40 cycles of 95°C for 1 minute, 58°C for 10 seconds, and 75°C for 30 seconds; extension at 75°C for 5 minutes, followed by a 15°C hold. The 382 bp amplicons were detected and visualized as described above.

Contamination was controlled for all work by using barrier pipet tips. Lab benches were bleached after each extraction and prior to any PCR work. DNA extraction, PCR setup, and the addition of template was performed in separate rooms to minimize cross contamination. All plasmid work was done in a separate room as well. Possible contamination in extractions and PCRs was monitored through the inclusion of extraction controls for each extraction and no template control that went through the same mechanical manipulations as samples after every third sample on all PCRs.

## Competing interests

The authors declare that they have no competing interests.

## Authors' contributions

NRH: Developed and ran the HPV-32 specific PCR assay, did all statistical analysis, and prepared the manuscript.NH: Extracted the clinical samples and tested them via the PGMY and HPV-32 dot blot assay. JEC: Developed and validated the HPV-32 dot blot assay used at the comparison assay for the HPV-32 specific PCR. JL: Provided subjects and performed oral examinations of all subjects in the study. MEH: Responsible for obtaining funding for the project and guidance for its direction, and preparation of the manuscript. All authors have read and approved the final manuscript.

## References

[B1] de Villiers EM (1997). Papillomavirus and HPV typing. Clin Dermatol.

[B2] de Villiers EM, Weidauer H, Le JY, Neumann C, zur Hausen H (1986). Papilloma viruses in benign and malignant tumors of the mouth and upper respiratory tract. Laryngol Rhinol Otol (Stuttg).

[B3] Lorincz AT, Reid R, Jenson AB, Greenberg MD, Lancaster W, Kurman RJ (1992). Human papillomavirus infection of the cervix: relative risk associations of 15 common anogenital types. Obstet Gynecol.

[B4] Volter C, He Y, Delius H, Roy-Burman A, Greenspan JS, Greenspan D, de Villiers EM (1996). Novel HPV types present in oral papillomatous lesions from patients with HIV infection. Int J Cancer.

[B5] Cameron JE, Hagensee ME, Meyers C (2007). Chapter 7: Human Papillomavirus Infection and Disease in HIV+ Individual. Aids-Associated Viral Oncogenesis.

[B6] King MD, Reznik DA, O'Daniels CM, Larsen NM, Osterholt D, Blumberg HM (2002). Human papillomavirus-associated oral warts among human immunodeficiency virus-seropositive patients in the era of highly active antiretroviral therapy: an emerging infection. Clin Infect Dis.

[B7] Leigh J (2000). Oral warts rise dramatically with use of new agents in HIV. HIV Clin.

[B8] Greenspan D, Canchola AJ, MacPhail LA, Cheikh B, Greenspan JS (2001). Effect of highly active antiretroviral therapy on frequency of oral warts. Lancet.

[B9] Marais DJ, Sampson C, Jeftha A, Dhaya D, Passmore JA, Denny L, Rybicki EP, Walt E Van Der, Stephen LX, Williamson AL (2006). More men than women make mucosal IgA antibodies to Human papillomavirus type 16 (HPV-16) and HPV-18: a study of oral HPV and oral HPV antibodies in a normal healthy population. BMC Infect Dis.

[B10] Gravitt PE, Peyton CL, Apple RJ, Wheeler CM (1998). Genotyping of 27 human papillomavirus types by using L1 consensus PCR products by a single-hybridization, reverse line blot detection method. J Clin Microbiol.

[B11] Bulkmans NW, Bleeker MC, Berkhof J, Voorhorst FJ, Snijders PJ, Meijer CJ (2005). Prevalence of types 16 and 33 is increased in high-risk human papillomavirus positive women with cervical intraepithelial neoplasia grade 2 or worse. Int J Cancer.

[B12] Flores R, Papenfuss M, Klimecki WT, Giuliano AR (2006). Cross-sectional analysis of oncogenic HPV viral load and cervical intraepithelial neoplasia. Int J Cancer.

[B13] Liaw KL, Hildesheim A, Burk RD, Gravitt P, Wacholder S, Manos MM, Scott DR, Sherman ME, Kurman RJ, Glass AG (2001). A prospective study of human papillomavirus (HPV) type 16 DNA detection by polymerase chain reaction and its association with acquisition and persistence of other HPV types. J Infect Dis.

[B14] Andersson-Ellstrom A, Hagmar BM, Johansson B, Kalantari M, Warleby B, Forssman L (1996). Human papillomavirus deoxyribonucleic acid in cervix only detected in girls after coitus. Int J STD AIDS.

[B15] Arora R, Kumar A, Prusty BK, Kailash U, Batra S, Das BC (2005). Prevalence of high-risk human papillomavirus (HR-HPV) types 16 and 18 in healthy women with cytologically negative Pap smear. Eur J Obstet Gynecol Reprod Biol.

